# Verdinexor, a Selective Inhibitor of Nuclear Exportin 1, Inhibits the Proliferation and Migration of Esophageal Cancer via XPO1/c-Myc/FOSL1 Axis

**DOI:** 10.7150/ijbs.66612

**Published:** 2022-01-01

**Authors:** Ling Ou, Xinyou Wang, Shumin Cheng, Min Zhang, Ruiqin Cui, Chunxia Hu, Shiyi Liu, Qian Tang, Yuying Peng, Ruihuan Chai, Shouxia Xie, Shaoxiang Wang, Wei Huang, Xiao Wang

**Affiliations:** 1 Bacteriology & Antibacterial Resistance Surveillance Laboratory, Shenzhen Institute of Respiratory Diseases, Shenzhen People's Hospital (The Second Clinical Medical College, Jinan University; The First Affiliated Hospital, Southern University of Science and Technology), Shenzhen 518020, Guangdong, China.; 2 The First District of Gastrointestinal Surgery, Sixth Affiliated Hospital of Sun Yat-sen University, Guangzhou, Guangdong, China.; 3 Department of Pharmacy, Shenzhen People's Hospital (The Second Clinical Medical College, Jinan University; The First Affiliated Hospital, Southern University of Science and Technology), Shenzhen 518020, Guangdong, China.; 4 School of Pharmacy, Jinan University, Guangzhou 510630, Guangdong, China; 5 School of Pharmaceutical Sciences, Shenzhen University Health Science Center, Shenzhen University, Shenzhen 518000, Guangdong, China.; 6 School of Pharmaceutical Sciences (Shenzhen), Sun Yat-sen University, Shenzhen 518107, Guangdong, China.

**Keywords:** Verdinexor, XPO1, esophageal cancer, c-Myc, FOSL1

## Abstract

Esophageal carcinoma (EC) ranks sixth among cancers in mortality worldwide and effective drugs to reduce EC incidence and mortality are lacking. To explore potential anti-esophageal cancer drugs, we conducted drug screening and discovered that verdinexor, a selective inhibitor of nuclear exportin 1 (XPO1/CRM1), has anti-esophageal cancer effects both *in vivo* and *in vitro*. However, the mechanism and role of verdinexor in esophageal cancer remain unknown. In the present study, we observed that verdinexor inhibited the proliferation and migration of EC cells *in vitro* and suppressed tumor growth *in vivo*. Additionally, we found that verdinexor induced cleavage of PARP and downregulated XPO1, c-Myc, and FOSL1 expression. RNA-sequence analysis and protein-protein interaction (PPI) analysis revealed that verdinexor regulated the XPO1/c-Myc/FOSL1 axis. The results of immunoprecipitation and proximity ligation assays confirmed that verdinexor disrupted the interaction between XPO1 and c-Myc. Overexpression of c-Myc rescued the inhibition of cell proliferation and cell migration caused by verdinexor. Overexpressed FOSL1 restored the inhibited migration by verdinexor. Taken together, verdinexor inhibited cell proliferation and migration of esophageal cancer via XPO1/c-Myc/FOSL1 axis. Our findings provide a new option for the development of anti-esophageal cancer drugs.

## Introduction

Esophageal carcinoma is a deadly disease, ranking seventh in terms of incidence with 604,000 new cases and sixth in terms of mortality overall with 544,076 deaths in 2020, and Eastern Asia has the highest incidence among men and women 1. The burden of esophageal cancer morbidity and mortality is rapidly increasing. Currently, the most commonly used strategies are chemotherapy, surgery or radiotherapy. Chemotherapy may be beneficial for patients with advanced (metastatic or disseminated) and recurrent esophageal cancers. Nevertheless, chemotherapy remains challenging, and exploring effective new drugs to treat esophageal cancer is of great significance.

XPO1 is a critical mediator of nuclear export, which is essential for regulating the cell cycle and proliferation of normal and malignant tissues 2-4. Studies have found that XPO1 protein is elevated in many cancers, such as osteosarcoma, pancreatic cancer and ovarian cancer 5-7. The suppression of XPO1 is a potential target for many cancer therapeutic interventions. Verdinexor can inhibit the expression of XPO1, as a novel orally bioavailable selective nuclear export inhibitor (SINE), which was reported for the first time to reduce the replication of respiratory syncytial virus *in vitro* and inhibit influenza virus replication *in vitro* and *in vivo* 8-10. Recent research has revealed that verdinexor exhibits biological activity against canine melanoma cell lines and canine osteosarcoma cell lines 11, 12. According to a recent announcement, the U.S. Food and Drug Administration (FDA) has conditionally approved verdinexor tablets for the treatment of dogs with lymphoma 13.

In this study, we screened 854 compounds and discovered verdinexor (KPT-335) as a new anti-esophageal cancer drug *in vitro* and *in vivo*. However, the role and mechanism of verdinexor in esophageal cancer remain unknown. It is necessary to clarify the potential pharmacological effects of verdinexor and provide a theoretical basis for the development of new drugs against esophageal cancer.

## Material and Methods

### Specimens and patient samples analysis

Three clinical human esophageal tumor tissues and corresponding adjacent normal tissues were obtained from the Sixth Affiliated Hospital of Sun Yat-sen University. The patient's prior consent and approval from the Institutional Research Ethics Committee of the Sun Yat-sen University Cancer Center were obtained for research purposes. Gene expression data were obtained from the Cancer Genome Atlas (TCGA) and Genotype-Tissue Expression (GTEx). Expression analysis was performed using the GEPIA2 online tool (http://gepia2.cancer-pku.cn/#general).

### Cell culture and compounds

Esophageal cancer cell lines KYSE30, KYSE450, KYSE180, KYSE510 and human normal esophageal squamous epithelial cell line NE1 were bought from the Cell Bank of Chinese Academy of Sciences (Shanghai, China). All cells were authenticated by short tandem repeat (STR) DNA profiling. Cells were cultured in RPMI1640 medium supplemented with 10 % fetal bovine serum (FBS, Gibco, USA) and 1 % antibiotics (100 U/ml penicillin and 100 mg/ml streptomycin, Gibco, USA) at 37°C in a humidified atmosphere of 5 % CO_2_. A clinical compound library containing 854 compounds was purchased from Topscience Co. Ltd. (Shanghai, China) and listed in **Supplementary Table S1,** and 21 active compounds were shown in** Supplementary Table S2**. Verdinexor (KPT-335) was obtained from Topscience Co. Ltd. (Shanghai, China) for drug treatment *in vitro*, dissolved in dimethyl sulfoxide (DMSO) and stored at -80 °C.

### Cell proliferation and cell viability assay

Cell proliferation and viability was measured using the Cell Counting Kit-8 (CCK-8, Beyotime, China). Cells were seeded in 96-well plates at 5,000 cells/well and treated with 20 μ M compounds. After 48 h of treatment, 10 μ L CCK-8 were added in 96-well plates. After 2 h of incubation, the OD value at 450 nm was detected using a microplate reader (Cytation 3, Biotek, USA). The control of the OD value at 450 nm was as 100% viability.

### Colony formation assay

Cells were seeded in 6-well plates at a density of 500 cells/well. Cells were treated with the indicated concentrations of verdinexor. The medium was changed every 3 days. After 14 days of incubation, the cells were washed with phosphate buffered saline (PBS, Gibco, USA), fixed with methanol, and stained with crystal violet. The clones were photographed and counted.

### Wound healing assay

A wound-healing assay was performed to detect cell migration. Cells were seeded at a density of 12,000 cells/well in 96-well plates. Following 12 h of synchronization with 2 % FBS RPMI1640 medium, cells were scratched by Auto Scratch (BioTek, USA) and photographed using Cytation 3 (BioTek, USA). The cells were treated with the indicated concentrations of verdinexor. After 24 h of drug treatment, the cells were photographed and the wound width was analyzed using Cytation 3 (BioTek, USA).

### RNA-sequence (RNA-seq) analysis

Total RNA was extracted using the RNeasy Mini Kit (Qiagen, Germany) according to the manufacturer's instructions. RNA-seq analysis was performed by the Beijing Genomics Institute. Co., Ltd. (Wuhan, China). Differentially expressed genes (DEGs) were extracted with |log2 fold change (FC)| > 1 and false discovery rate (FDR) < 0.05. The results of the DEGs were displayed by a volcano plot. The PPI network of DEGs was performed using the Search Tool for the Retrieval of Interacting Genes (STRING) online analysis tool (https://string-db.org/cgi/input.pl).

### Reverse transcription-quantitative PCR (RT-qPCR)

Total RNA was isolated using the RNeasy Mini Kit (Qiagen, Germany) according to the manufacturer's instructions. RNA was reverse transcribed to cDNA using Prime Script RT Master Mix (Takara, Japan). CDNA (100 ng) was detected by qRT-PCR using TB Green® (Takara, Japan) in Light Cycler 480 System (Roche, Switzerland). Primers were synthesized by BBI Life Sciences and listed in** Supplementary Table S3**.

### Western blot analysis

Cells were treated with drugs in 6-well plates, washed twice with PBS, and lysed in radioimmunoprecipitation assay (RIPA) buffer (Beyotime, China) containing a protease inhibitor cocktail and phosSTOP phosphatase inhibitor Cocktail (Roche, Switzerland) for 30 min on ice. Lysis buffer was collected and centrifugated at 12,500 rpm for 15 min. The protein concentration of cell lysates was evaluated using the BCA Protein Assay Kit (Beyotime, China). The lysates were boiled for 10 min at 100 °C after mixing with SDS-loading buffer (Beyotime, China). Equal amounts of protein were loaded on SDS-PAGE gels from 8 % to 12 % and transferred to PVDF membranes (Millipore, USA). The membranes were blocked in 5% skimmed milk for 1 h at room temperature, washed three times with Tris-buffered saline containing 0.1 % Tween 20 (TBST), and incubated with the indicated antibodies overnight at 4 °C. Membranes were washed three times with TBST and incubated with anti-rabbit or anti-mouse IgG for 1 h at room temperature. The membranes were visualized using enhanced chemiluminescence (Millipore, USA) and imaged using the ChemiDoc Touch Imaging System (Bio-Rad, USA). GAPDH, β-actin, or β-tubulin was used as loading control.

### RNA interference

Cells were transfected with small interfering RNAs (siRNAs) using Lipofectamine RNAimax (Invitrogen, USA) following the manufacturer's instructions. All siRNAs were purchased from GenePharma (Shanghai, China). Negative control siRNAs were labeled as NC. The sequences of the siRNAs are listed in **Supplementary Table S4**.

### Plasmid transfection

Cells were transfected with plasmids using Lipofectamine 3000 (Invitrogen, CA, USA) according to the manufacturer's instructions.

### Immunoprecipitation (IP)

After 24 h drug treatment, cells were lysed with IP buffer containing protease and phosphatase inhibitors. Beads were washed with IP wash buffer five times before use. First, the cell lysates were incubated with beads for 3 h. After adsorption using a magnetic stand, the cell supernatant was collected and incubated with XPO1 (Cell Signaling Technology, #46249, 1:50) or c-Myc (Cell Signaling Technology, #18583, 1:50) at 4 °C overnight. Next, the cell supernatant was incubated with beads for 6 h and the pellet was collected. The pellet was mixed with load buffer and boiled at 100 °C for 10 min. The samples were analyzed using western blotting.

### Immunofluorescence

Cells were washed in PBS, fixed in 4 % paraformaldehyde for 15 min, and permeabilized with 0.1 % Triton X-100 for 5 min. Samples were blocked in 5 % bovine serum albumin and incubated with primary antibodies overnight at 4 °C. The stained cells were washed twice with PBS and incubated with Alexa Fluor-conjugated secondary antibodies at room temperature for 1 h in darkness. Additionally, the cells were incubated with DAPI for 10 min in darkness. Images were captured with a Zeiss LSM510 Meta confocal microscope (Carl Zeiss, Jena, Germany).

### Drug Affinity Responsive Target Stability (DARTS) assay

Cell lysates were obtained from the KYSE30 cells. Cells were scraped and lysed with HEPES lysis buffer containing a protease inhibitor and a phosphatase inhibitor. After centrifugation at 12,000 × g for 15 min, the protein concentration was quantified using the BCA Protein Assay Kit (Beyotime, China). Before drug treatment, the protein concentration was diluted to 5 mg/mL. Then, the cell lysates were treated with the verdinexor and DMSO for 3 h at 37 ℃. Samples were incubated with pronase (Roche, Switzerland, 1 μg pronase with 2 mg proteins, 1:2000) or distilled water for 20 min at 37 ℃. After protease digestion, the candidate target proteins were detected by western blot analysis. Β-Actin was used as an internal control.

### The Duo link *in situ* proximity ligation assay (PLA)

The Duo link *in situ* proximity ligation assay (PLA) was performed according to the manufacturer's protocol (Sigma-Aldrich). The antibodies used for PLA were XPO1 (Cell Signaling Technology, #46249, 1:200) and c-Myc (Cell Signaling Technology, #18583, 1:200). Duo links and DAPI signals were detected using a confocal microscopy.

### Nude mice xenograft tumor models

Five-week-old male nude mice (BABL/c, n = 12) were obtained from Guangdong Medical Laboratory Animal Center (Foshan, Guangdong, China) and housed at the Institute of Laboratory Animal Science of Jinan University (SPF grade). All animal experiments were approved by the Institutional Animal Care and Use Committee of Jinan University. KYSE 30 cells (2×10^6^) were suspended in PBS: Matrigel at a ratio of 2:1 and injected into the left or right flank of mice. After the tumors reached approximately 100 mm^3^, mice were administered with vehicle and verdinexor (5 mg/kg, dissolved in 2 % DMSO, 40 % PEG300, 5 % Tween-80 and 53 % saline) via intraperitoneal injection. Tumor size was measured by calipers every other day using the following formula:

Volume = ½ × larger diameter × (smaller diameter) ^2^

After 24 days, the mice were sacrificed, and tumors were carefully removed, photographed and weighed immediately.

### Immunohistochemistry (IHC)

Paraffin-embedded sections were treated with xylene twice for 10 min each, rehydrated gradually with ethanol ranging from 100 % to 70 % and finally immersed in distilled water. The slides were permeabilized with 0.3 % hydrogen peroxide, followed by antigen retrieval by heating the specimens in sodium citrate buffer. After blocking with 10 % goat serum, the slides were incubated with primary antibody overnight and then incubated with the corresponding secondary antibody for 30 min. The sections were further incubated with 3, 3′-diaminobenzidine (DAB) (ZSGB-BIO, Beijing, China) and counterstained with 10 % hematoxylin.

### Statistical analysis

Data are presented as the mean ± standard deviation (SD) and statistically analyzed using the Student's t-test (two-sided) or one-way ANOVA. Tukey's post-hoc comparison was used to determine the statistical differences between the treatment groups. Differences were considered statistically significant and results are shown as *P < 0.05, **P < 0.01 and not significant (N.S.).

## Results

### A novel XPO1 inhibitor has anti-esophageal cancer activity and XPO1 is highly expressed in esophageal squamous cancer

**Figure 1:**
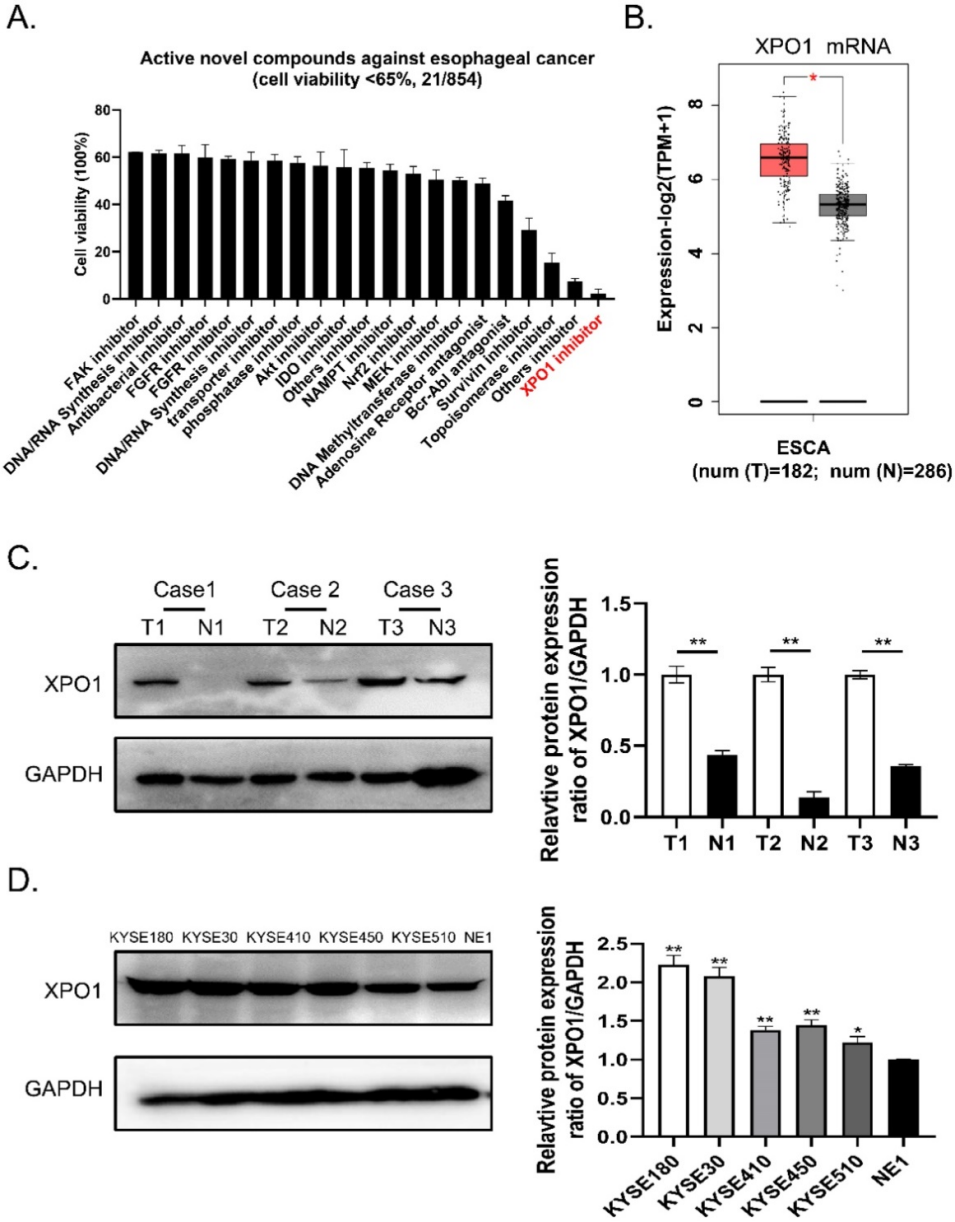
** A novel XPO1 inhibitor have anti-esophageal cancer activity and XPO1 is highly expressed in esophageal squamous cancer (A)** Cell viability of 21 active compounds against esophageal cancer from clinical compound library. The XPO1 inhibitor verdinexor was identified as an active drug. Cells were seeded in 96-well plates and treated with 20 μM drugs for 48 h and cell viability was measured by CCK-8 assay. **(B)** XPO1 gene expression from TCGA database and GTEx database analyzed by the GEPIA 2 online tool. **(C)** Representative western blot analyses XPO1 protein expression in three cases of esophageal carcinoma and normal esophageal tissues. **(D)** Representative western blot analyses XPO1 protein expression in esophageal squamous cells (KYSE30, KYSE450, KYSE180, KYSE410, and KYSE510) and normal esophageal cells (NE1). The data was analyzed by Student's t-test (two-sided) or one-way ANOVA. Error bars represent ± SD, *P < 0.05, **P < 0.01.

To identify potential small compounds against esophageal squamous cancer, we screened 854 clinical compound libraries using the Cell Counting Kit-8 assay (**Supplementary Table S1**). We discovered 21 active novel compounds against esophageal cancer and identified a novel XPO1 inhibitor verdinexor as a new targeted anti-esophageal cancer drug (**Figure 1A, Supplementary Table S2**). Therefore, we conducted differential gene expression analysis based on the TCGA and GTEx databases and found XPO1 mRNA is highly expressed in esophageal squamous cancer compared with normal tissue samples (**Figure 1B**). Furthermore, we investigated the expression of XPO1 in three cases of esophageal carcinoma and normal esophageal tissues. As shown in** Figure 1C**, XPO1 was highly expressed in esophageal squamous cancer. In addition, XPO1 expression was over-expressed in esophageal squamous cells compared to that in normal esophageal cells (**Figure 1D**).

### Verdinexor inhibited cell proliferation in esophageal cancer

**Figure 2:**
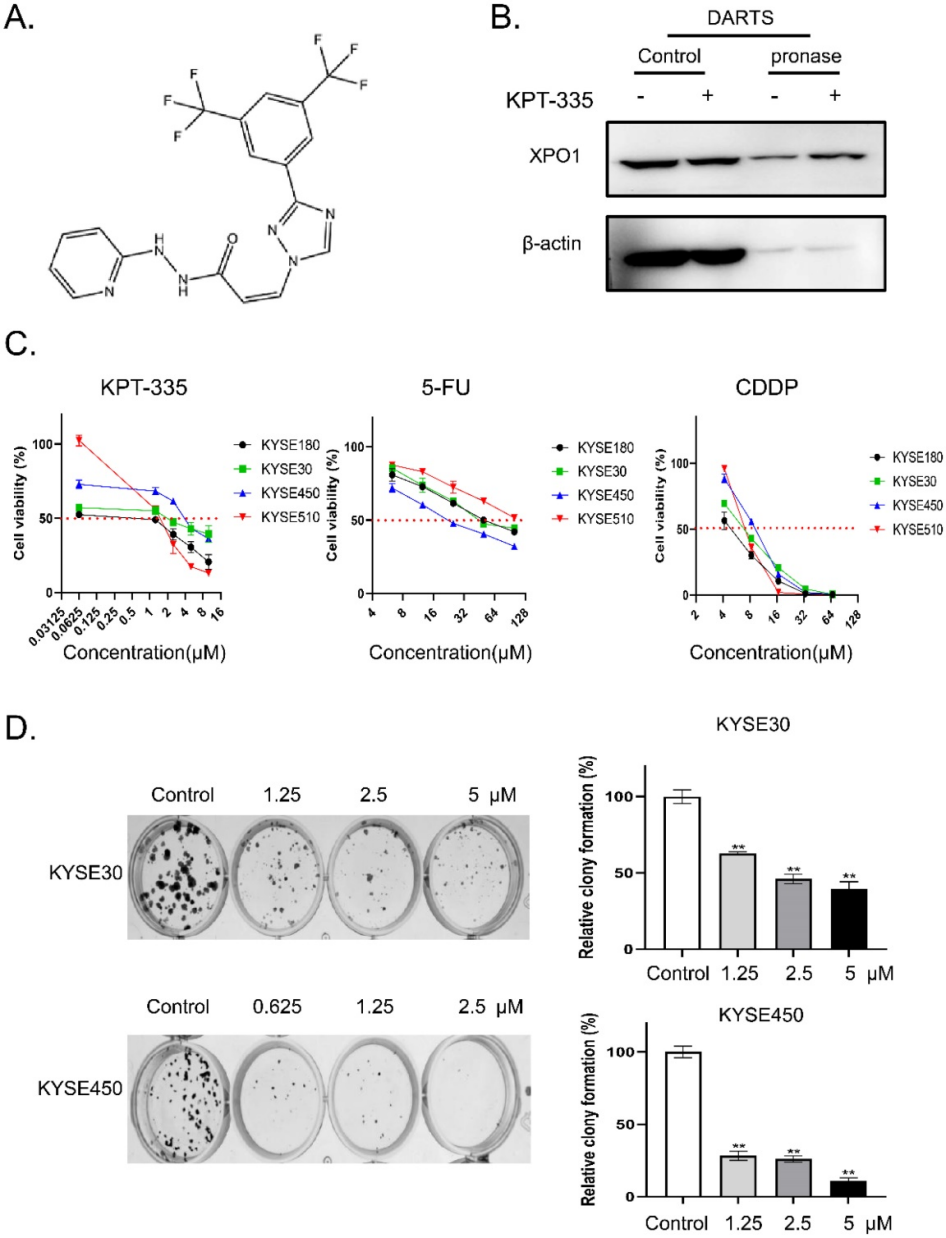
** Verdinexor could bind to XPO1 protein and inhibited cell proliferation in esophageal cancer. (A)** Chemical structure of verdinexor. **(B)** The DARTS assay for target validation. XPO1 protein stability was increased upon KPT-335 (100 μM) treatment in KYSE30 lysates. Pronase treatment was conducted for 20 min. **(C)** The IC_50_ of verdinexor, cisplatin and 5-fluorouracil for 48 h was performed by CCK-8 assay in esophageal squamous cancer cells (KYSE30, KYSE450, KYSE180 and KYSE510). **(D)** Verdinexor inhibited the colony formation ability of KYSE30 and KYSE450. The data were analyzed by Student's t-test (two-sided) or one-way ANOVA. Error bars represent ± SD, *P < 0.05, **P < 0.01.

The chemical structure of verdinexor was shown in **Figure 2A**. The binding of verdinexor to XPO1 was verified by the DARTS assay (**Figure 2B**). As shown in **Figure 2C** and **Table 1**, the IC_50_ of verdinexor was lower than that of cisplatin (CDDP) and 5-fluorouracil (5-Fu) in esophageal squamous cancer cells. Meanwhile, verdinexor inhibited the colony formation in esophageal squamous cancer cells (**Figure 2D**). These results demonstrated that verdinexor may act as a novel drug against cell growth in esophageal cancer.

### Verdinexor suppressed migration, induced cleavage of PARP and downregulated XPO1 expression in esophageal squamous cancer

**Figure 3:**
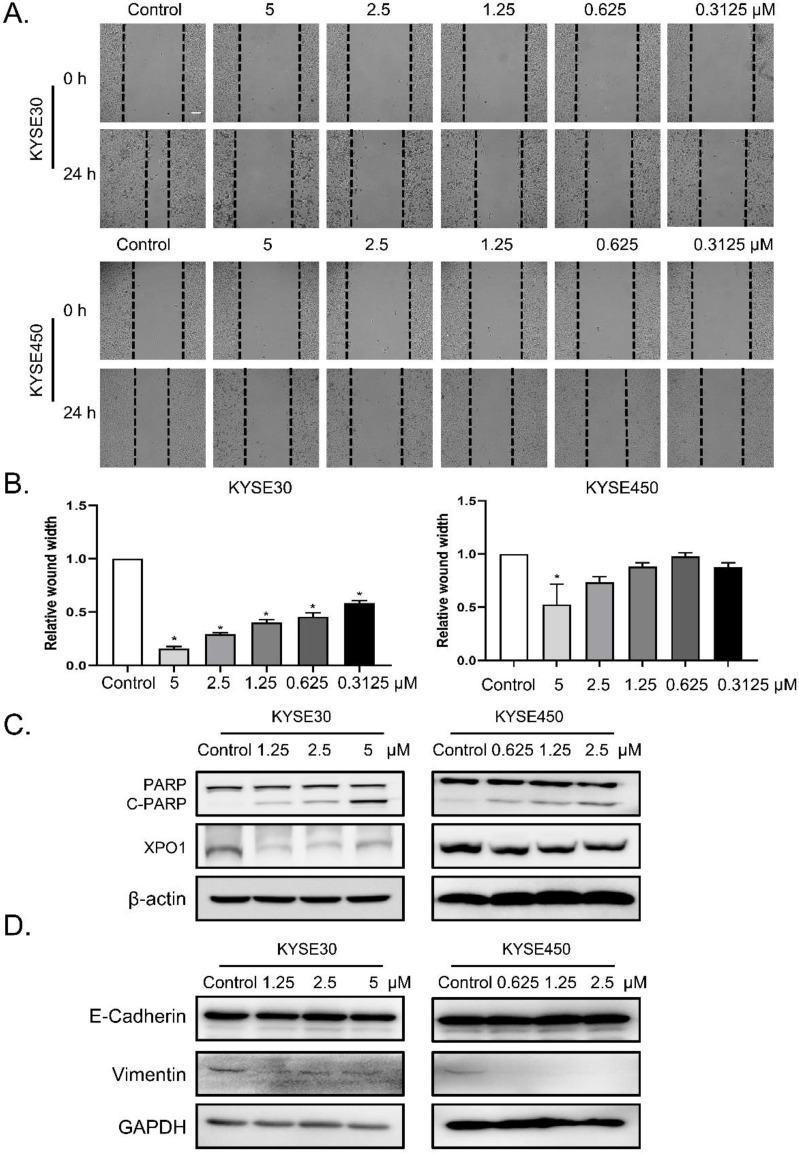
** Verdinexor suppressed migration, induced cleavage of PARP and downregulated XPO1 expression in esophageal squamous cancer.** KYSE30 and KYSE450 cells were treated with the indicated concentrations of verdinexor for 24 h. Wound width was shown by representative images **(A)** and the ratio of wound width **(B)** was analyzed by wound migration assay. Scale bars, 100 µm. **(C&D)** XPO1, PARP and cleaved PARP, E-cadherin and Vimentin protein expression were detected by western blot assay. The data were analyzed by Student's t-test (two-sided) or one-way ANOVA. Error bars represent ± SD, *P < 0.05, **P < 0.01.

To determine the role of verdinexor in esophageal squamous cancer, we performed migration assays and found that the wound width rate was suppressed by verdinexor (**Figure 3A and 3B**). Before wound healing assay, cells were treated with KPT-335 for 24 h and cell viability was determined by using CCK-8 assay. The results were shown in **Figure S1** and we found that the cell survival of concentration of KPT-335 (from 0.3125 to 5 μ M) for 24 h was exceeded 75%, indicating that KPT-335 is almost non-toxic under the condition of no more than 5 μ M for 24 h. Western blot analysis showed that verdinexor activated the cleavage of poly (ADP-ribose) polymerase (PARP) and significantly inhibited XPO1 expression **(Figure 3C)**. Additionally, the migration marker, vimentin, was suppressed by verdinexor (**Figure 3D**).

### Verdinexor inhibited XPO1/c-Myc /FOSL1 axis in esophageal cancer

**Figure 4:**
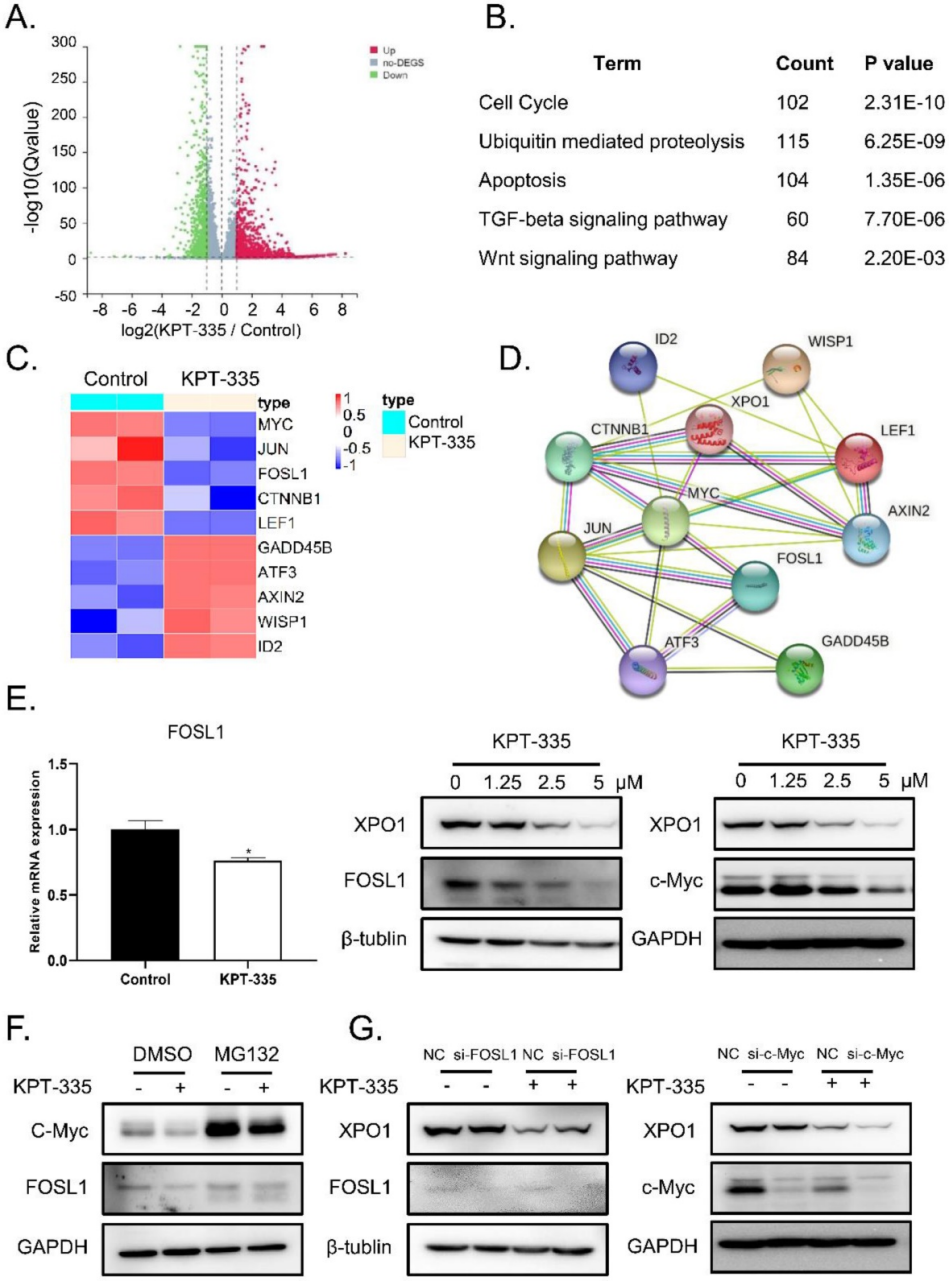
** Verdinexor inhibited XPO1/c-Myc/FOSL1 axis.** KYSE30 cells were treated with verdinexor for 24 h. **(A)** Differential genes by RNA-sequence analysis were shown by a volcano map. **(B)** KEGG pathway enrichment analysis were shown with *P* < 0.05. **(C)** Differential genes related with Wnt pathway were shown in a heat map. **(D)** The interaction of XPO1 and related Wnt pathway differential genes was displayed by PPI network. **(E)** The FOSL1 gene expression was analyzed by RT-qPCR and protein expression of XPO1, FOSL1 and c-Myc were detected by western blot assay. **(F)** KYSE30 cells were treated with verdinexor, MG132 or a combination of verdinexor and MG132 for 8 h. FOSL1 and c-Myc expressions were detected by western blot assay. **(G)** KYSE30 cells were transfected with small RNA of FOSL1 or c-Myc for 24 h and then treated with verdinexor for 24 h. FOSL1, c-Myc and XPO1 expressions were analyzed by western blot assay. The data were analyzed by Student's t-test (two-sided) or one-way ANOVA. Error bars represent ± SD, *P < 0.05, **P < 0.01.

To identify the mechanism of verdinexor in esophageal squamous cancer, we performed RNA-sequence analysis after treatment with verdinexor in KYSE30 cells. Consequently, 4709 down-regulated differential genes and 3767 up-regulated differential genes are shown in a volcano map (**Figure 4A**). KEGG pathway enrichment analysis showed that verdinexor mainly acts on the Wnt pathway, cell cycle, apoptosis, ubiquitin mediated proteolysis, and TGF-beta signaling pathways (**Figure 4B**). Differential gene heat map results showed that verdinexor significantly downregulated the transcription factors c-Myc and FOSL1 located downstream in the Wnt pathway (**Figure 4C**). As shown in **Figure 4D**, the related Wnt pathway differential genes were displayed by the PPI network. Inhibition of XPO1 by verdinexor significantly downregulated FOSL1 expression. Meanwhile, western blot analysis showed that verdinexor suppressed the XPO1/c-Myc/FOSL1 axis (**Figure 4E**). As shown in **Figure 4F**, the downregulation of c-Myc induced by verdinexor could be reversed by MG132, which is an inhibitor of ubiquitination, while that of FOSL1 cannot. In addition, knockdown of FOSL1 or c-Myc using small RNA alone did not affect XPO1 expression, while the combination of c-Myc knockdown and verdinexor treatment could significantly inhibit XPO1 expression (**Figure 4G**).

### Inhibition of c-Myc /FOSL1 enhanced the activity of verdinexor

**Figure 5:**
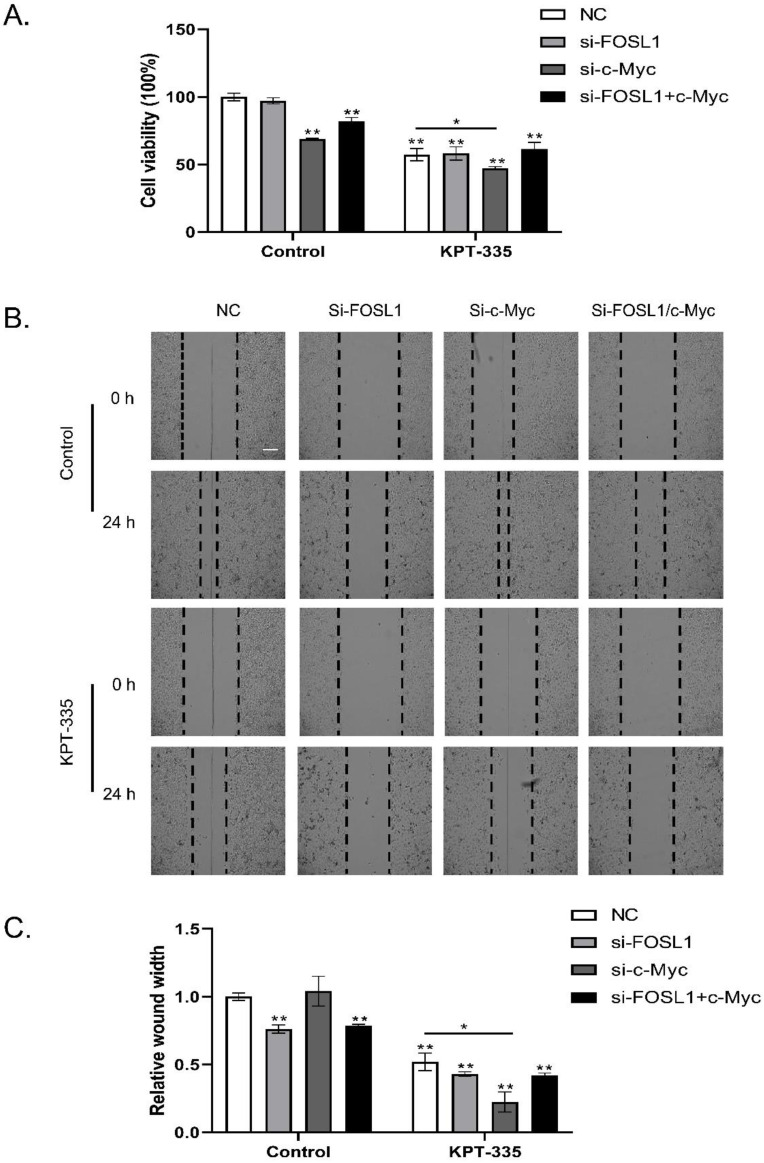
** Inhibition of c-Myc/FOSL1 enhanced the activity of verdinexor.** KYSE30 cells were transfected with small RNA of FOSL1, c-Myc or both for 24 h and then treated with verdinexor for 24 h. **(A)** Cell viability was analyzed by CCK-8 assay. Wound width was shown by representative images** (B)** and the ratio of wound width **(C)** was analyzed by wound healing assay. Scale bars, 100 µm. The data were analyzed by Student's t-test (two-sided) or one-way ANOVA. Error bars represent ± SD, *P < 0.05, **P < 0.01.

As shown in **Figure 5A**, knockdown of c-Myc alone or c-Myc/FOSL1 inhibited cell growth, whereas knockdown of FOSL1 alone did not. Silencing c-Myc enhanced verdinexor-inhibited cell growth. Knockdown of FOSL1 alone or FOSL1/c-Myc significantly inhibited the wound width rate, whereas knockdown of c-Myc alone could not. Knockdown of c-Myc enhanced the inhibition of verdinexor (**Figure 5B and 5C**).

### Overexpression of c-Myc rescued verdinexor-suppressed cell proliferation and overexpressed c-Myc or FOSL1 restored the inhibited-migration caused by verdinexor

**Figure 6:**
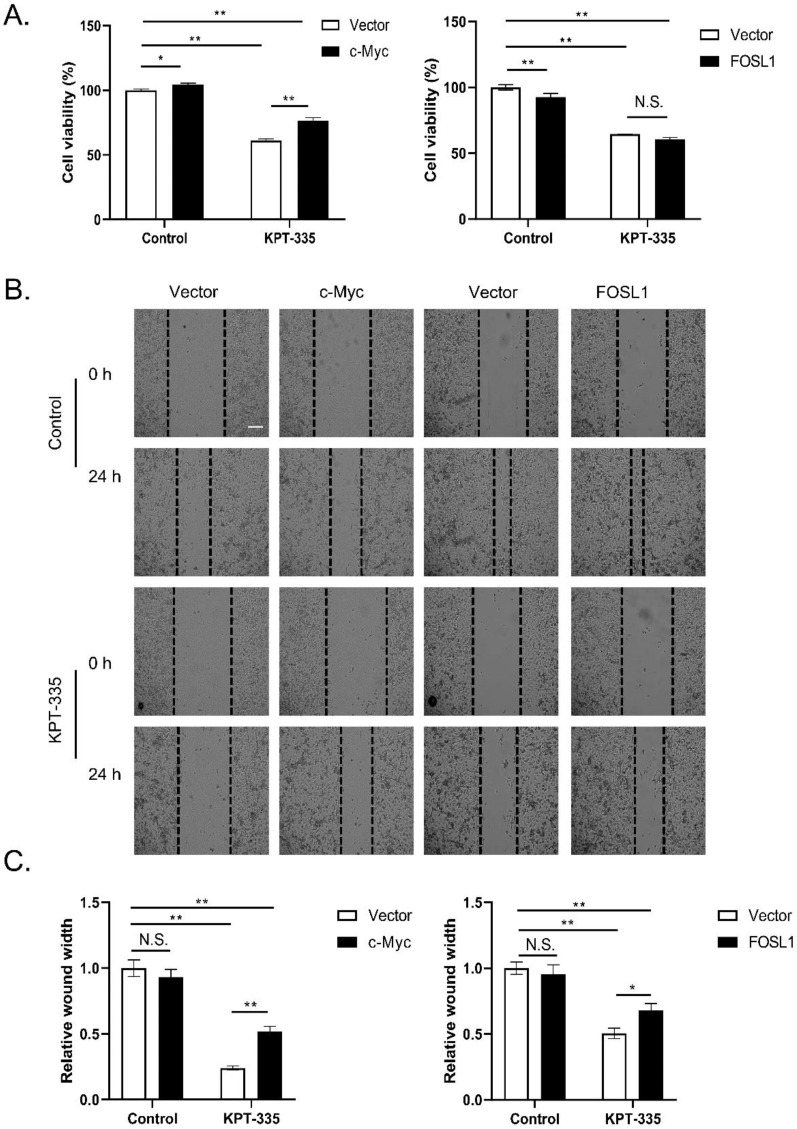
** Overexpression of c-Myc rescued verdinexor--suppressed cell proliferation and overexpressed c-Myc or FOSL1 restored inhibited- migration caused by verdinexor.** KYSE30 cells were transfected with plasmid of FOSL1 or c-Myc for 24 h then treated with verdinexor for 24 h. **(A)** Cell viability was analyzed by CCK-8 assay. Wound width was shown using representative images **(B)** and the ratio of wound width **(C)** was analyzed by wound healing assay. Scale bars, 100 µm. The data were analyzed by Student's t-test (two-sided) or one-way ANOVA. Error bars represent ± SD, *P < 0.05, **P < 0.01.

To determine the role of c-Myc/FOSL1 in verdinexor-suppressed cell proliferation, we transfected the plasmid c-Myc or FOSL1 into cells and then treated them with verdinexor. As presented in **Figure 6A**, overexpression of c-Myc reversed verdinexor-suppressed cell proliferation, whereas FOSL1 did not. Overexpression of c-Myc or FOSL1 rescued verdinexor-inhibited cell migration** (Figure 6B and 6C)**. Taken together, FOSL1 only plays a key role in verdinexor-induced cell migration, and c-Myc functions as a crucial regulator of cell proliferation and migration caused by verdinexor.

### Verdinexor significantly inhibited nuclear accumulation of c-Myc

**Figure 7:**
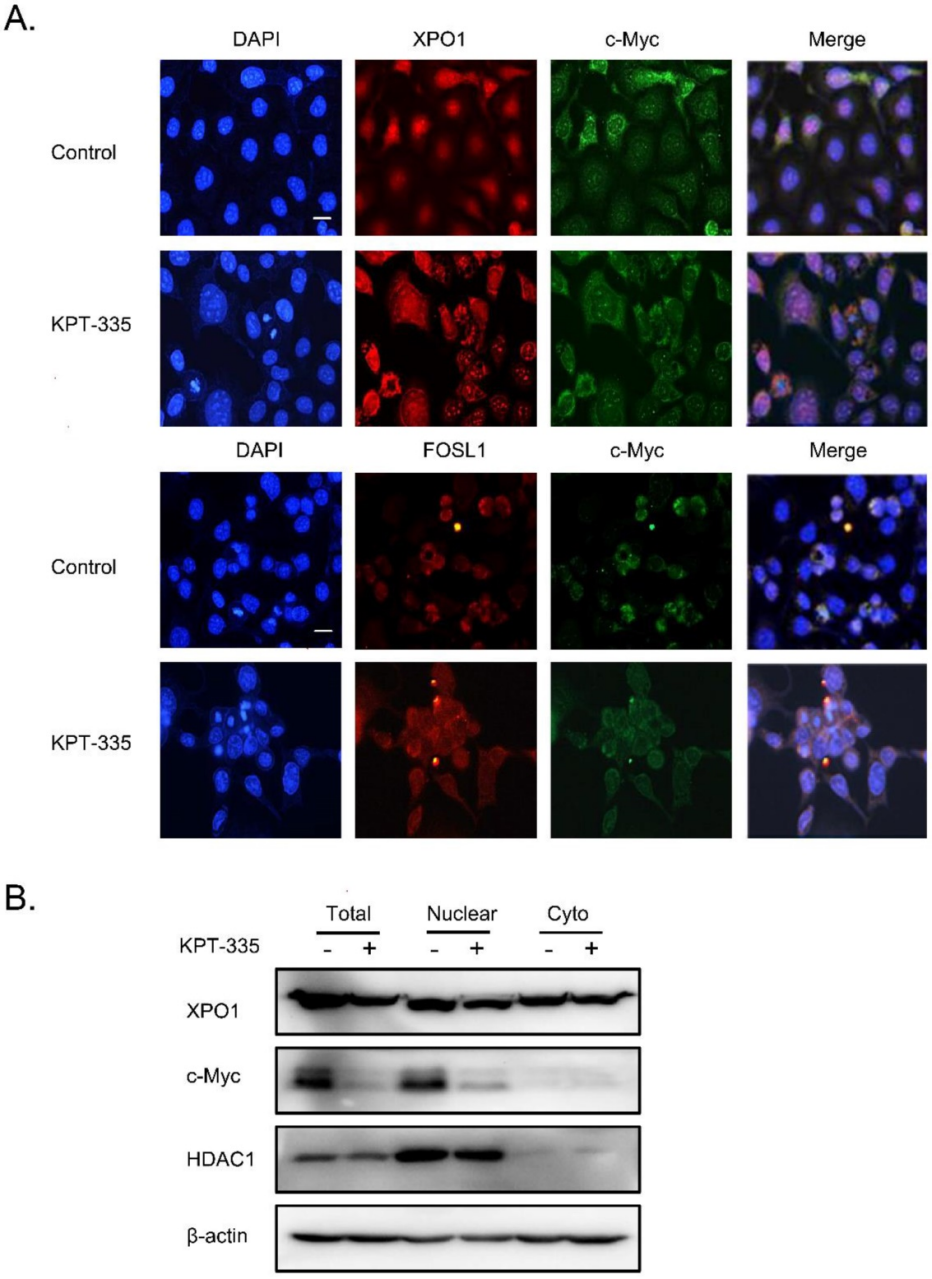
** Verdinexor significantly inhibited nuclear accumulation of c-Myc.** KYSE30 cells were treated with verdinexor for 24 h. **(A)** the colocalization experiment was using immunofluorescence to detect the colocalization of XPO1, c-Myc and FOSL1. Scale bars, 100 µm.** (B)** Cell fractionation experiments were performed and the expressions of XPO1 and c-Myc in the nucleus and cytoplasm (Cyto) were analyzed by western blot assay.

To further explore the role of XPO1/c-Myc/FOSL1 axis in verdinexor-inhibited cell survival, we performed a colocalization experiment using immunofluorescence, and the results showed that c-Myc, XPO1 and FOSL1 were mainly located in the nucleus (**Figure 7A**). Cell fractionation experiments demonstrated that verdinexor suppressed XPO1 in the cytoplasm and nucleus, and significantly inhibited the expression of c-Myc in the nucleus (**Figure 7B**).

### Verdinexor disturbed the interaction between XPO1 and c-Myc

**Figure 8:**
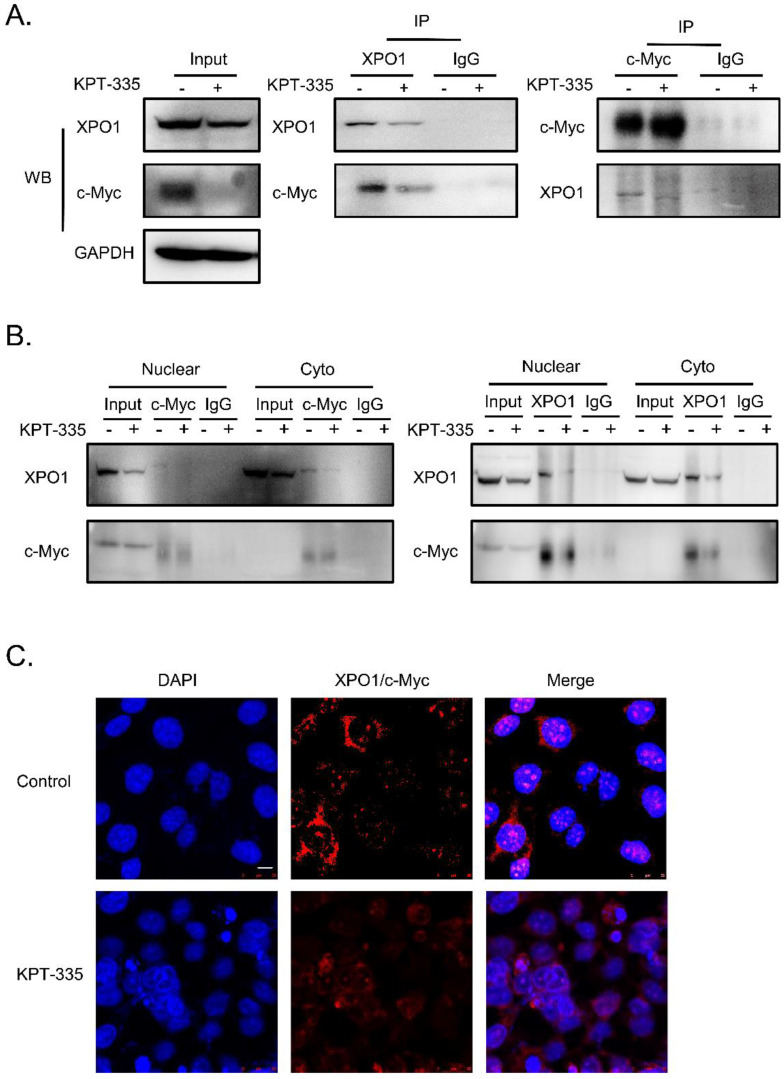
** Verdinexor disturbed the interaction between XPO1 and c-Myc.** KYSE30 cells were treated with verdinexor for 24 h. **(A)** the interaction of XPO1 and c-Myc was detected by immunoprecipitation analysis and western blot assay. **(B)** Cell fractionation experiments were conducted and the interaction of XPO1 and c-Myc in the nucleus and cytoplasm was detected by immunoprecipitation analysis and western blot assay. **(C)** The interaction of XPO1 and c-Myc was detected by proximity ligation assay. Scale bars, 100 µm.

To further determine the interaction of XPO1/c-Myc, we performed IP analysis and the results showed that the interaction between XPO1 and c-Myc could be disrupted by verdinexor (**Figure 8A and 8B**). In addition, to validate the interaction of the XPO1/c-Myc axis, we detected the interaction of the two proteins using the proximity ligation assay. Similarly, verdinexor disturbed the interaction between XPO1 and c-Myc (**Figure 8C**).

### Verdinexor inhibited tumor growth and suppressed XPO1 and c-Myc expression *in vivo*

**Figure 9:**
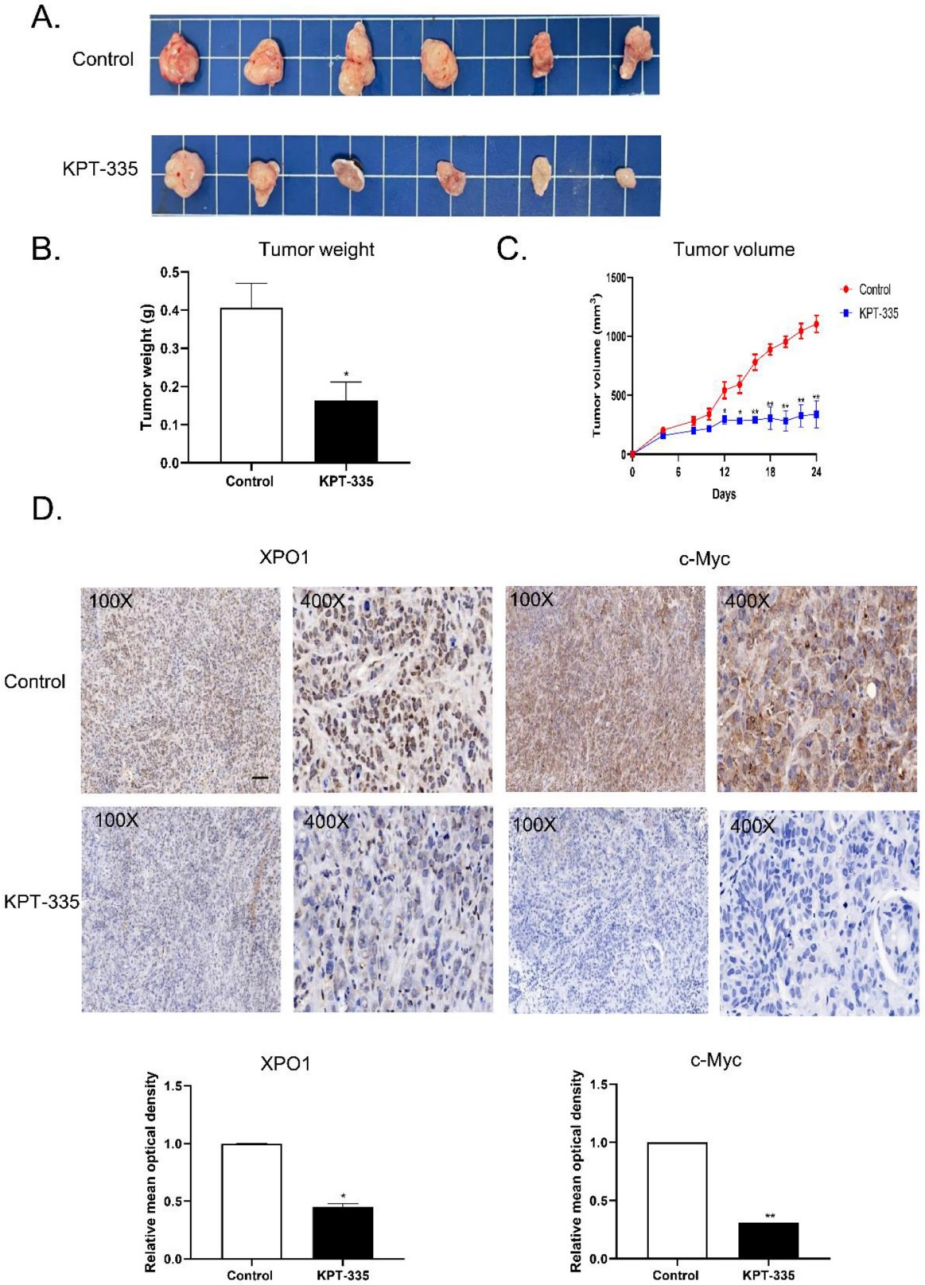
** Verdinexor inhibited tumor growth and suppressed XPO1 and c-Myc expression *in vivo*. (A)** KYSE30 cells were implanted in nude mice, treated with control and verdinexor (5 mg/kg, dissolved in 2 % DMSO, 40 % PEG300, 5 % Tween-80 and 53 % saline). The images showed the relative size of the tumors. Scale bars, 1 cm. **(B&C)** The tumor volumes were monitored every other day, and once the mice were euthanized, the tumor was weighed immediately. **(D)** XPO1 and c-Myc expressions were analyzed by immunohistochemistry. Scale bars, 100 µm. The data were analyzed by Student's t-test (two-sided) or one-way ANOVA. Error bars represent ± SD, *P < 0.05, **P < 0.01.

To validate the effect of verdinexor* in vivo*, we tested verdinexor in nude mice xenograft models. The tumor volume of the treated group was significantly reduced, compared to that of the control group (**Figure 9A**). Tumor weight and tumor volume showed a similar decreasing trend; the inhibition of tumor rate was 59.5% (**Figure 9B**). IHC analysis showed that after verdinexor treatment, the number of XPO1- and c-Myc-positive cells were significantly reduced (**Figure 9C and 9D**). In summary, verdinexor significantly inhibited tumor growth *in vivo*.

## Discussion

Effective clinical therapy for esophageal carcinoma remains an unsolved challenge. Through drug screening, we found that a novel small molecule inhibitor, verdinexor, has anti-esophageal cancer activity. Herein, we demonstrated that verdinexor inhibits the cell survival and migration of esophageal carcinoma cells and suppresses tumor growth *in vivo*. Moreover, verdinexor binds to XPO1 and inhibits the interaction between XPO1 and c-Myc. Verdinexor inhibits cell survival and migration of esophageal carcinoma via the XPO1/c-Myc/FOSL1 axis.

Nuclear import and export are strictly controlled in cancer, and cancer cells are thought to be susceptible to nuclear transport inhibition 14, 15. Hence, inhibition of the nuclear transport system has the potential to develop targeted drugs to treat cancer 16. Birnbaum reported that XPO1 serves as a marker of poor prognosis in pancreatic adenocarcinoma 17. Lin *et al.* found that XPO1 can be used as a drug candidate owing to the gene mutation and protein overexpression of XPO1 in esophageal cancer 18. Similarly, in this study, we found that XPO1 mRNA was overexpressed in esophageal carcinoma compared to normal samples. We also illustrated that XPO1 was significantly increased in ESCA patient tissues compared to that in adjacent tissues.

In this study, we first proved that inhibition of XPO1 has an anti-esophageal cancer effect. The Darts assay confirmed that verdinexor can bind to XPO1. Western blotting showed that verdinexor can inhibit XPO1 and c-Myc. c-Myc may play a crucial role in verdinexor-suppressed cell proliferation. Interestingly, RNA-seq KEGG pathway analysis revealed that verdinexor regulates the Wnt pathway. C-Myc and FOSL1 were identified as targets of Wnt/β-catenin signaling and c-Myc is the major downstream effector of the Wnt pathway 19,20. C-Myc is a proto-oncogene and its expression level is closely related to cell proliferation in cancer 21, 22. Overexpression of c-Myc leads to differences in gene family expression levels, which leads to increased cell proliferation 23. FOSL1 as an oncogene promotes the progression of most malignant tumors 24. Recent studies have shown that FOSL1 accelerates the metastasis of head and neck squamous cell carcinoma, and FOSL1 stimulates the metastasis and tumorigenesis of colorectal cancer 25, 26. RNA-seq analysis showed that verdinexor significantly inhibits c-Myc and FOSL1, and western blot results proved that verdinexor inhibits both. These results are consistent with previous studies showing that selective inhibitors of nuclear export (SINE) XPO1 antagonists such as KPT-276, KPT-330, and KPT-185 can downregulate c-Myc expression in cancer 27-29. Therefore, we attempted to determine the relationship between XPO1, c-Myc, and FOSL1. Previous study has revealed that XPO1 is positively regulated by c-Myc 30. Similarly, we found that knockdown of c-Myc by small RNA alone cannot affect XPO1 expression, whereas knockdown of c-Myc followed by treatment with verdinexor can significantly decrease XPO1 and inhibit cell viability and wound width. Inhibition of FOSL1 did not affect XPO1, but enhanced the reduced cell migration of verdinexor. This suggests that FOSL1 is not dependent on XPO1, but rather on c-Myc. Emerging evidence suggests that serine/threonine kinase can be recruited to the E-boxes of FOSL1 by c-Myc 31. DEG protein-protein interaction (PPI) analysis illustrated that the XPO1/c-Myc/FOSL1 axis is modulated by verdinexor.

The XPO1/c-Myc/FOSL1 axis may act as a key regulator in verdinexor-inhibited cell migration. c-Myc amplification may serve as a potential biomarker for ESCC^32^. The dysregulation of c-Myc plays a key role in cell proliferation and migration. Overexpression of c-Myc rescued cell proliferation and cell migration induced by verdinexor. Abnormal nuclear or cytoplasmic localization of transcription factors can affect tumor progression 33,34. C-Myc has a specific region that can be targeted into the nucleus 35. Nuclear localization of c-Myc can target DNA sequences to regulate cell growth and proliferation 36. Geisler reported that nuclear and cytoplasmic c-Myc staining was an important factor in predicting survival in endometrial carcinoma, and patients whose tumors stained positively for nuclear c-Myc and negatively for cytoplasmic c-Myc had significantly worse survival 37. Further analysis indicated that c-Myc, as an oncogene, was mainly located in the nucleus. Our results showed that verdinexor inhibited c-Myc expression in the nucleus and disrupted the interaction between XPO1 and c-Myc. It suggests that verdinexor may be more suitable for those with positive expression of c-Myc in the nucleus.

## Conclusions

In conclusion, verdinexor inhibits the proliferation and migration of esophageal cancer via the XPO1/c-Myc/FOSL1 axis. This provides a new strategy for the development of new anti-esophageal cancer drugs.

## Supplementary Material

Supplementary table 1.

Supplementary tables 2-4.

Supplementary figure.

## Figures and Tables

**Table 1:** The IC_50_ of verdinexor, cisplatin and 5- fluorouracil.

IC_50_ (μM)	KPT-335	CDDP		5-FU
KYSE180	1.45 ± 0.01		3.71 ± 0.03	54.68 ± 0.58
KYSE 30	2.58 ± 0.16		5.89 ± 0.23	56.62 ± 0.47
KYSE450	3.88 ± 0.16		7.82 ± 0.01	24.33 ± 2.19
KYSE510	1.44 ± 0.17		6.05 ± 0.24	108.3 ± 1.13
				

## Abbreviations

EC: Esophageal carcinoma
XPO1/CRM1: exportin 1
PPI: protein-protein interaction
SINE: selective inhibitor of nuclear export
FDA: Food and Drug Administration
TCGA: the Cancer Genome Atlas
GTEx: Genotype-Tissue Expression
STR: short tandem repeat
FBS: fetal bovine serum
DMSO: dimethyl sulfoxide
CCK-8: Cell Counting Kit-8
PBS: phosphate buffered saline
RNA-seq: RNA-sequence
DEGs: Differentially expressed genes
FC: fold change
FDR: false discovery rate
STRING: Search Tool for the Retrieval of Interacting Genes
RT-qPCR: Reverse transcription-quantitative PCR
RIPA: radioimmunoprecipitation assay
TBST: Tris-buffered saline containing 0.1 % Tween 20
siRNAs: small interfering RNAs
NC: Negative control
IP: Immunoprecipitation
DARTS: Drug Affinity Responsive Target Stability
PLA: proximity ligation assay
IHC: Immunohistochemistry
DAB: 3, 3′-diaminobenzidine
SD: standard deviation
N.S.: not significant
CDDP: cisplatin
5-Fu: 5-fluorouracil
PARP: poly (ADP-ribose) polymerase
