# Supplementary material for: Verdinexor, a Selective Inhibitor of Nuclear Exportin 1, Inhibits the Proliferation and Migration of Esophageal Cancer via XPO1/c-Myc/FOSL1 Axis
Source: Int J Biol Sci. 2022 Jan 1;18(1):276–91. doi: 10.7150/ijbs.66612 (PMC8692140; doi:10.7150/ijbs.66612)
Supplement: Supplementary file 1 — Supplementary table 1. [file ijbsv18p0276s1.pdf]

Supplementary Table S1. Clinical compound library.

Number	Name
1	Defactinib (VS-6063, PF-04554878)
2	PX-12
3	Enasidenib
4	CL-387785 (EKI-785)
5	EVP-6124 hydrochloride
6	Epirubicin hydrochloride
7	BIIB-021
8	LXR623
9	D-Pantethine
10	Eucalyptol
11	(-)-Cotinine
12	Dixanthogen
13	Guaiacol
14	Eugenol
15	Gabexate mesylate
16	Idebenone
17	Ellagic acid
18	Cinchonine
19	Taurine
20	Oltipraz

21	Nefiracetam
22	Docetaxel trihydrate
23	Fluticasone propionate
24	Prednisolone phosphate sodium
25	Loperamide hydrochloride
26	Levodropropizine
27	Foscarnet sodium
28	Lobeline hydrochloride
29	Sodium salicylate
30	Bisoctrizole
31	Isovaleramide
32	Cytisine
33	Tolperisone hydrochloride
34	Indigo
35	Paeoniflorin
36	Cloperastine hydrochloride
37	Succinic acid
38	Daidzein
39	Noscapine hydrochloride
40	Carbenoxolone disodium
41	D-Biotin
42	Lonidamine

43	Nonivamide
44	Diatrizoate sodium
45	Hydroquinidine
46	Tetracycline
47	Allantoin
48	Epiandrosterone
49	Cyproterone acetate
50	Trimebutine maleate
51	Chlorophyllin
52	Ciprofibrate
53	Diacerein
54	Piceid
55	Adenosine disodium triphosphate
56	Arecoline HBr
57	(S)-(+)-Camptothecin
58	Cortodoxone
59	Cortisone
60	Spermine
61	Kynurenine
62	Azobenzene
63	Ursolic acid
64	Luteolin

65	Brivudine
66	Gallamine triethiodide
67	Piroctone olamine
68	Tetrabenazine
69	Allopurinol
70	Rifampicin
71	Troxerutin
72	Serotonin hydrochloride
73	Honokiol
74	Gastrodin
75	Curcumin
76	Cordycepin
77	Pinocembrin
78	Tetrandrine
79	5-hydroxytryptophan (5-HTP)
80	Rotundine, TETRAHYDROPALMATINE HYDROCHLORIDE
81	Naringenin
82	Baicalin
83	Andrographolide
84	Osthole
85	Caffeic Acid
86	Phlorizin

87	Tanshinone IIA
88	Cantharidin
89	Emodin
90	Pterostilbene
91	(+)-Catechin Hydrate
92	Trimebutine
93	Ebselen
94	Pregnenolone
95	Noradrenaline bitartrate
96	Quercetin
97	Artemisinin
98	Quinacrine dihydrochloride
99	Acetylleucine
30	Sodium butanoate
101	Nilvadipine
102	AICAR (Acadesine)
103	Promestriene
104	Pirenzepine dihydrochloride
105	ADP
106	Y-27632 dihydrochloride
107	SB 431542
108	Adenosine 5'-monophosphate

109	Semicarbazide hydrochloride
110	Genistein
111	Lumacaftor(VX 809)
112	Indole-3-carbinol
113	TG100-115
114	BAY 87-2243
115	Doxifluridine
116	Deferoxamine Mesylate
117	N-Acetylneuraminic acid
118	CNV-1014802
119	LY344864
120	Vipadenant
121	LY2835219
122	MK1775
123	Irbinitinib(ARRY-380; ONT-380)
124	ZK-811752, BX-471
125	Pilaralisib (XL147)
126	GSK923295
127	RGB-286638 free base
128	Danusertib (PHA-739358)
129	EPZ6438
130	CYT997 (Lexibulin)

131	GSK-2636771
132	APD 668
133	DOV 21947 hydrochloride(Amitifadine hydrochloride)
134	PQ401
135	Emapunil
136	CUDC-907
137	XL147 analogue
138	CEP-32496
139	LCL161
140	INK 128 (MLN0128)
141	SB 742457
142	BX795
143	Eniporide
144	BX912
145	AM281
146	MLN8237(Alisertib)
147	VER-49009
148	VX661
149	KenPaullone
150	BMS-833923
151	RU58841
152	CX5461

153	SGX-523
154	HA14-1
155	SD-208
156	Anacetrapib (MK-0859)
157	ABT-737
158	Ganetespib (STA-9090)
159	YM155
160	PHA-793887
161	ABT888 hydrochloride
162	(+)-JQ-1
163	NVP-BEP800
164	Piboserod(SB-207266)
165	ABT-263 (Navitoclax)
166	Triapine
167	SB715992(Ispinesib)
168	AEE788
169	MPI-0479605
170	SB743921
171	PSI6206
172	PLX-3397
173	Fexinidazole
174	Nandrolone

175	Thymopentin
176	Cilastatin
177	VX-702
178	WHI-P154
179	Torcetrapib
180	MK5046
181	BEZ235 (NVP-BEZ235, Dactolisib)
182	Cariporide
183	Losmapimod (GW856553X)
184	CX-4945 (Silmitasertib)
185	RU 24969 hemisuccinate
186	Endoxifen
187	BLU9931
188	LY-2874455
189	TAK875
190	ENMD-2076
191	CEP-28122
192	PCI-24781 (Abexinostat)
193	Decernotinib(VX-509)
194	Dacomitinib (PF299804, PF299)
195	TAS-301
196	Palomid 529 (P529)

197	TAK901
198	GTS 21 dihydrochloride
199	HA130
200	AP26113
201	Fedratinib (SAR302503, TG101348)
202	Neratinib(HKI-272)
203	Kaempferol
204	PF 573228
205	Ilomastat (GM6001, Galardin)
206	Ciproxifan maleate
207	Pelitinib (EKB-569)
208	ARN-509
209	Notoginsenoside R1
210	Ginsenoside Rg1
211	Stevioside
212	Shikonin
213	Ginsenoside Rb1
214	Gossypol acetic acid
215	Ginsenoside Re
216	Icariin
217	Rosmarinic acid
218	Chlorogenic Acid

219	Schisandrin
220	Curcumol
221	Forskolin
222	Asiaticoside
223	Borneol
224	Monocrotaline
225	Harmine hydrochloride
226	Suramin Sodium Salt
227	Glucosamine sulfate
228	Triptolide (PG490)
229	Pimasertib (AS-703026)
230	Foretinib (GSK1363089)
231	NS 1619
232	Alvelestat (AZD9668)
233	PFK-158
234	Testosterone Enanthate
235	Potassium canrenoate
236	LY2608204
237	Atopaxar Hydrobromide
238	CUDC-101
239	2-Amino-6-mercaptopurine
240	Selumetinib (AZD6244)

241	Entinostat (MS-275)
242	PD0325901
243	Saracatinib (AZD0530)
244	DAPT (GSI-IX)
245	BI 2536
246	Iniparib (BSI-201)
247	Rolipram
248	MLN4924
249	GKT137831
250	D-(+)-Trehalose dihydrate
251	TSU-68 (SU6668, Orantinib)
252	LY2835219 mesylate
253	Ropinirole hydrochloride
254	Obatoclax Mesylate (GX15-070)
255	Silymarin
256	Protoporphyrin IX
257	Salubrinal
258	Vincamine
259	Ginkgolide A
260	Ginkgolide C
261	Ginkgolide B
262	Clinafloxacin

263	Bilobalide
264	Otenabant (CP-945598) hydrochloride
265	Balicatib
266	AZD1080
267	NSI-189
268	Momelotinib (CYT387)
269	BKM120 (NVP-BKM120, Buparlisib)
270	SAR245409 (XL765)
271	Picropodophyllin (PPP)
272	Clemizole
273	Apitolisib (GDC-0980, RG7422)
274	MK3697
275	Dinaciclib (SCH727965)
276	Afuresertib
277	MBX2982
278	Methazolamide
279	Acalisib (GS-9820)
280	PF-04217903
281	OC000459
282	Crenolanib
283	Tivozanib (AV-951)
284	Perifosine (KRX-0401)

285	Dapivirine (TMC120)
286	NLG919
287	Tiplaxtinin(PAI-039)
288	A-803467
289	Bohemine
290	WHI-P180
291	AVN944
292	Apilimod
293	AM095
294	LY294002
295	Omecamtiv mecarbil (CK-1827452)
296	TP0903
297	GDC-0032
298	Ponalrestat
299	CHS 828
300	GDC-0941
301	Mertansine
302	LY2090314
303	ABT-751 (E7010)
304	Pirodavir
305	OTSSP167
306	TAK-063

307	Eliprodil
308	VX-680 (Tozasertib, MK-0457)
309	Linifanib (ABT-869)
310	MK-8776 (SCH 900776)
311	RVX-208
312	MPEP hydrochloride
313	Azeliragon
314	SB-505124
315	PHT-427
316	KNK437
317	PNU 282987
318	Lomeguatrib
319	R406 free base
320	HMN214
321	Veliparib (ABT-888)
322	Canertinib (CI-1033)
323	PF-562271
324	Binimetinib (MEK162, ARRY-162, ARRY-438162)
325	Ascomycin (FK520)
326	MK-2866 (GTx-024)
327	UNBS-5162
328	Galunisertib (LY2157299)

329	AZD5438
330	Semaxanib (SU5416)
331	SRT1720
332	PLX4720
333	PAC1
334	Mocetinostat (MGCD0103)
335	Roxadustat (FG-4592)
336	Plinabulin (NPI-2358)
337	Andarine
338	BMS-777607
339	JNJ-1661010
340	GSK-2256098
341	SB590885
342	Roscovitine (CYC202,Seliciclib)
343	LB-100
344	Quizartinib (AC220)
345	Semaxinib(SU 5416)
346	Nimorazole
347	ACT 058362; Palosuran
348	Altiratinib(DCC2701)
349	ADX 47273
350	AZD1208

351	TAE684 (NVP-TAE684)
352	Clemizole hydrochloride
353	AG-1478
354	Pritelivir (BAY 57-1293)
355	Amuvatinib(MP470)
356	CPI-203
357	AZD2461
358	CC-292 (AVL-292)
359	Brivanib Alaninate (BMS-582664)
360	Osu-03012
361	ARRY380
362	PD184352 (CI-1040)
363	FG-2216
364	AZ5104
365	PRT062607 (P505-15, BIIB057) hydrochloride
366	Avasimibe
367	GSK1292263
368	AT13148
369	IOWH-032
370	STF-31
371	BMS-754807
372	KW2449

373	Glycopyrrolate
374	Tideglusib
375	TG101209
376	AT9283
377	Fenretinide
378	AR-A014418
379	Luminespib (AUY-922, NVP-AUY922)
380	GSK2126458(Omipalisib)
381	AZD8055
382	AWD 131-138
383	R788 (Fostamatinib) Disodium
384	MSDC-0160
385	Almorexant
386	SNS-314 Mesylate
387	MK0752
388	LGK-974
389	CH5132799
390	SGI-1776 free base
391	GLPG0634 analogue
392	FK866 (APO866, Daporinad)
393	Vilanterol (GW642444; GW 642444X)
394	Gandotinib (LY2784544)

395	LY2811376
396	G-749
397	INCB024360 analogue
398	DCC-2036 (Rebastinib)
399	Rostafuroxin(PST 2238)
400	Poziotinib (HM781-36B)
401	MI-2 (Menin-MLL Inhibitor)
402	MK-8245
403	WAY-100635 Maleate
404	BAY85-3934;Molidustat
405	XL019
406	OSI 930
407	Flavopiridol (Alvocidib) hydrochloride
408	Fruquintinib
409	Elacridar
410	WHI-P131
411	Varespladib (LY315920)
412	Barasertib (AZD1152-HQPA)
413	AZD7545
414	Vidofludimus(4SC-101; SC12267)
415	JNJ-26854165 (Serdemetan)
416	JNJ-7777120

417	AZD4547
418	GSK3787
419	MK-2206 dihydrochloride
420	SB225002
421	AZD2858
422	AZD2014
423	ASP 3026
424	INCB28060(Capmatinib)
425	BYL-719(Alpelisib)
426	Filgotinib (GLPG0634)
427	EPZ005687
428	AZD-5363
429	GSK-525762A(I-BET-762)
430	PH797804
431	BGJ398 (NVP-BGJ398)
432	Gedatolisib (PF-05212384, PKI-587)
433	AT 7519 hydrochloride salt
434	Erastin
435	Ro 48-8071 fumarate
436	CHIR-99021
437	Loxiglumide
438	Nutlin-3

439	WP1066
440	Ampalex
441	Lu-28-179
442	ATB 346
443	TAK632
444	Ro 61-8048
445	CI994 (Tacedinaline)
446	XAV939
447	Pracinostat (SB939)
448	P7C3
449	Cabotegravir (GSK744, GSK1265744)
450	CP-466722
451	AMG-337
452	Duvelisib (IPI-145, INK1197)
453	NS-398
454	XL388
455	CH5183284 (Debio-1347)
456	PF-2545920
457	BIBS 39
458	MG-132
459	JNJ-42165279
460	Verdinexor (KPT-335)

461	AZD-3965
462	PF06463922
463	Ro 46-2005
464	SB203580
465	CW069
466	EPZ015666
467	F 11440
468	CVT-313
469	DAA1106
470	Belnacasan (VX-765)
471	Motesanib (AMG-706)
472	R112
473	Nutlin-3a
474	Taprenepag
475	Napabucasin
476	Briciclib
477	APD597
478	Tivantinib (ARQ 197)
479	Oprozomib (ONX 0912)
480	Daprodustat
481	L-α-Phosphatidylcholine
482	Lanolin

483	Acetovanillone
484	PKC-412(Midostaurin)
485	Betaine
486	Metadoxine
487	Propidium Iodide
488	Lysozyme
489	N-Sulfo-glucosamine sodium salt
490	Breviscapin
491	ALPHA-BOSWELLIC ACID
492	(R)-(+)-Limonene
493	Perillyl alcohol
494	Canertinib dihydrochloride
495	Parthenolide
496	Velpatasvir
497	Combretastatin A4
498	Verbascoside
499	Rigosertib (ON-01910)
500	BAF312 (Siponimod)
501	PD123319
502	Azd6738
503	GDC-0994
504	NXY-059

505	AG-14361
506	Sotrastaurin
507	Elesclomol (STA-4783)
508	Ponesimod
509	Levodropropizine
510	Isosteviol
511	Calcium carbonate
512	Xanthohumol
513	Geldanamycin
514	Licochalcone A
515	Cinobufagin
516	CC-122
517	LX-4211
518	Demethylzeylasteral
519	PF-04418948
520	Baohuoside I
521	Syringin
522	Icaritin
523	Brigatinib
524	Epacadostat
525	Solcitinib
526	LEE011

527	RG2833 (RGFP109)
528	Ensartinib
529	GSK-2881078
530	Cutamesine dihydrochloride
531	Sodium Dichloroacetate
532	Mubritinib (TAK 165)
533	Chelerythrine chloride
534	SB705498
535	Trichostatin A (TSA)
536	AMG-517
537	Evofosfamide
538	COH29
539	GSK2879552
540	INO-1001
541	ZSTK474
542	CEP-18770 (Delanzomib)
543	Acalabrutinib
544	PS-1145
545	ETC-1002
546	BFH772
547	Fostamatinib (R788)
548	MK571

549	Relugolix
550	MLN1117
551	Doravirine
552	LY3039478
553	HTHQ
554	Avitinib maleate
555	AZD3759
556	HMN-176
557	BGP-15
558	VTP-27999
559	Picropodophyllin (PPP)
560	Secoisolariciresinol diglucoside
561	CDDO(Bardoxolone)
562	L-Mimosine
563	Quercetin Dihydrate
564	Tolcapone
565	Icotinib
566	Ruboxistaurin hydrochloride
567	Nepicastat (SYN-117) HCl
568	PA-824
569	VX-745
570	GW 501516

571	Darapladib (SB-480848)
572	Beta-Lapachone
573	EX 527 (Selisistat)
574	URB597
575	EHT 1864
576	R406
577	A 740003
578	LY2228820
579	Heparin sodium
580	Entrectinib
581	XL413 hydrochloride
582	S49076
583	THZ1
584	NU2058
585	Bardoxolone Methyl
586	LFM-A13
587	TDZD-8
588	Staurosporine
589	SU11274
590	Volasertib (BI 6727)
591	AZD8931 (Sapitinib)
592	WP1130

593	OSI-906 (Linsitinib)
594	MLN8054
595	LY2603618
596	PF-477736
597	AT406 (SM-406, ARRY-334543)
598	CC-223
599	Idasanutlin (RG-7388)
600	BIA10-2474
601	ABT-639
602	TRX818
603	ACY-241
604	Avitinib
605	PF-3274167
606	Selonsertib
607	Pepstatin
608	SCH900776 S-isomer
609	Lasmiditan
610	GDC-0084
611	Acebilustat
612	Birinapant
613	E-64
614	Turofexorate Isopropyl (XL335)

615	Epothilone B (EPO906, Patupilone)
616	Tipifarnib
617	SNX-2112 (PF-04928473)
618	Calpeptin
619	Embelin
620	Galeterone
621	MK-886 (L-663,536)
622	T0901317
623	AZD7762
624	PF-04691502
625	ODM-201
626	Rapastinel
627	PF-4136309
628	Erdafitinib
629	BAY1217389
630	Merimepodib
631	UK-371804
632	Bay 41-4109 (racemate)
633	S107
634	(±)-Equol
635	Ezutromid
636	Clindamycin

637	NMS-P937 (NMS1286937)
638	Salirasib
639	VER-49009
640	AT7519
641	KD025 (SLx-2119)
642	Ipatasertib (GDC-0068)
643	R428 (BGB324)
644	Tirasemtiv
645	VX-222 (VCH-222, Lomibuvir)
646	BAY 80-6946 (Copanlisib)
647	TAK-733
648	MK-4827(Niraparib) tosylate
649	Uprosertib (GSK2141795)
650	AST-1306
651	AT-406
652	SRT2104 (GSK2245840)
653	Tepotinib (EMD 1214063)
654	BTZ043
655	AZD6482
656	SF2523
657	DMXAA (Vadimezan)
658	Guanosine

659	LY2801653
660	Talazoparib (BMN 673)
661	Gemcitabine HCl
662	CC-115
663	GS-9620
664	Exp3174
665	Olumacostat Glasaretil
666	LY2886721
667	Verubecestat (MK-8931)
668	NQ301
669	Ozanimod (RPC1063)
670	ARQ-092
671	Compound 4
672	Itacitinib
673	EW-7197
674	Verinurad
675	Olmutinib (HM61713, BI 1482694)
676	RG 13022
677	RG 14620
678	ACY-738
679	PBTZ169
680	GAL-021

681	ORY-1001 (RG-6016)
682	CB-839
683	Lu AF21934
684	Mivebresib
685	NS 638
686	EGF816
687	Marimastat(BB-2516)
688	CB-5083
689	SCH58261
690	AZD8186
691	AZD9496
692	Fosbretabulin (Combretastatin A4 Phosphate (CA4P)) Disodium
693	Enzastaurin (LY317615)
694	BLU-554
695	Efaproxiral Sodium
696	BAY-876
697	AZD0156
698	CPI-1189
699	eFT508
700	AZD5153
701	Lonafarnib
702	PD168393

703	OTX015
704	Dalcetrapib (JTT-705, RO4607381)
705	CX691
706	TMS
707	BIRB 796 (Doramapimod)
708	TAK-715
709	tak-013
710	Calcitriol
711	Telatinib
712	SNS-032 (BMS-387032)
713	Triciribine
714	PI-103
715	SU9516
716	CHIR-124
717	Semagacestat (LY450139)
718	WAY 200070
719	Pamapimod (R-1503, Ro4402257)
720	Monomethyl auristatin E (MMAE)
721	NVP-AEW541
722	PHA-665752
723	Tasisulam
724	Equol

725	Palmitoylethanolamide
726	Halofuginone hydrobromide
727	Halobetasol Propionate
728	PX-478 2HCl
729	(+)-Usniacin
730	AZD1981
731	Lvguidingan
732	AS1842856
733	SC1
734	ADL5859 HCl
735	Bafetinib (INNO-406)
736	Fostemsavir
737	Ulixertinib (BVD-523, VRT752271)
738	Apilimod mesylate
739	PCI-27483
740	Fevipiprant
741	GDC-0152
742	Quisinostat (JNJ-26481585)
743	MGCD-265
744	Aloxistatin
745	KX2-391
746	JI-101

747	ABC294640
748	MK-2461
749	AZD1283
750	MGL3196
751	Valrocemide
752	BLZ945
753	NIH-12848
754	IMD 0354
755	HSP990 (NVP-HSP990)
756	CP-724714
757	Solasonine
758	Solamargine
759	orcinol
760	phenylalanine
761	LY3023414
762	Phorbol
763	Thymoquinon
764	CX-6258
765	Cadazolid
766	Nolatrexed
767	Avibactam
768	Betahistine

769	Amenamevir
770	PF-3758309 hydrochloride
771	Anlotinib Dihydrochloride
772	DEXTRORPHAN D-TARTRATE
773	AN2718
774	AR-42
775	Selinexor (KPT-330)
776	MK-5108 (VX-689)
777	CPI-203
778	SAR-20347
779	TAK-659 (hydrochloride)
780	Tasquinimod
781	SMI-4a
782	Givinostat (ITF2357)
783	AS-8351
784	Pacritinib (SB1518)
785	PCI-34051
786	SB1317(TG-02) hydrochloride
787	C29
788	MLN2480
789	BGT226 (NVP-BGT226)
790	OTS514

791	OTS964
792	ON123300
793	PLX8394
794	AZD8835
795	LY3214996
796	Vatalanib (PTK787) 2HCl
797	Dovitinib (TKI-258, CHIR-258)
798	BX-517
799	Refametinib (RDEA119, Bay 86-9766)
800	Golvatinib (E7050)
801	PF-00562271
802	PHA767491 HCl
803	Indoximod (NLG-8189）
804	Amcasertib
805	Relebactam
806	N6022
807	Tenatoprazole
808	Epetraborole hydrochloride
809	TRx0237 (LMTX) mesylate
810	BMS-986020
811	S1RA hydrochloride
812	Ocaperidona

813	Batimastat (BB-94)
814	Bindarit
815	AHU377
816	ZM241385
817	Exendin-4
818	VLX1570
819	PF-06282999
820	Ledipasvir (acetone)
821	Resiquimod
822	Bosentan
823	Talabostat mesylate
824	L755507
825	CGS 27023A
826	AZD3759 hydrochloride
827	GLPG1690
828	ETC-159
829	FGF-401
830	NCB-0846
831	Sulfatinib
832	Anavex 2-73
833	AGI 1067
834	Tempol

835	Laquinimod
836	Emricasan
837	Repertaxin
838	Eltrombopag Olamine
839	Pyridoxal phosphate
840	AFN-1252
841	LY404039
842	VCMMAE
843	Dasotraline hydrochloride
844	MD 39-AM
845	Acelarin
846	RO4929097
847	S-38093
848	4-Oxofenretinide
849	Setipiprant
850	ly2334737
851	XL-652
852	RG-7112
853	E 6005; RVT-501
854	Octanoic acid（CPI-613）
