# Supplementary material for: Verdinexor, a Selective Inhibitor of Nuclear Exportin 1, Inhibits the Proliferation and Migration of Esophageal Cancer via XPO1/c-Myc/FOSL1 Axis
Source: Int J Biol Sci. 2022 Jan 1;18(1):276–91. doi: 10.7150/ijbs.66612 (PMC8692140; doi:10.7150/ijbs.66612)
Supplement: Supplementary file 2 — Supplementary tables 2-4. [file ijbsv18p0276s2.pdf]

Supplementary Table S2. Active compounds against esophageal cancer.

Number	Name	Target	Cell Viability (100%)
**1**	Defactinib (VS-6063)	FAK inhibitor	62.19
**166**	Triapine	DNA/RNA Synthesis inhibitor	61.49
**808**	Epetraborole hydrochloride	Antibacterial inhibitor	61.49
**188**	LY-2874455	FGFR inhibitor; VEGFR inhibitor	59.76
**455**	CH5183284 (Debio-1347)	FGFR inhibitor	59.07
**845**	Acelarin	DNA/RNA Synthesis inhibitor	58.55
**370**	STF-31	transporter inhibitor	58.47
**325**	Ascomycin (FK520)	phosphatase inhibitor	57.66
**276**	Afuresertib	Akt inhibitor	56.27
**286**	NLG919	IDO inhibitor	55.62
**783**	AS-8351	Others inhibitor	55.46
**299**	CHS 828	NAMPT inhibitor	54.51
**561**	CDDO (Bardoxolone)	Nrf2 inhibitor	53.02
**240**	Selumetinib (AZD6244)	MEK inhibitor	50.41
**239**	2-Amino-6-mercaptopurin	DNA Methyltransferase inhibitor	50.31

	e		
**271**	Picropodophyllin (PPP)	Adenosine Receptor antagonist;<br>Aurora Kinase inhibitor	48.98
**254**	Obatoclax Mesylate<br>(GX15-070)	Bcr-Abl antagonist	41.69
**159**	YM155	Survivin inhibitor	29.28
**6**	Epirubicin hydrochloride	Topoisomerase inhibitor	15.27
**726**	Halofuginone<br>hydrobromide	Others inhibitor	7.37
**460**	Verdinexor<br>(KPT-335)	XPO1 inhibitor	2.30

Supplementary Table S3. Primers used for qPCR analyses.

Gene name	Sequences	
	Forward Primer	Reverse Primer
FOSL1	CAGGCGGAGACTGACAAACTG	TCCTTCCGGGATTTTGCAGAT
GAPDH	GGAGCGAGATCCCTCCAAAAT	GGCTGTTGTCATACTTCTCATGG

Supplementary Table S4. The sequences of siRNAs used in knockdown experiments.

Gene name	Sequences	
	sense（5'-3'）	antisense（5'-3'）
FOSL1-homo-1210	CUAGCACAAUUUGCACUAATT	UUAGUGCAAAUUGUGCUAGTT
FOSL1-homo-301	GCCAAGCAUCAACACCAUGTT	CAUGGUGUUGAUGCUUGGCTT
FOSL1-homo-926	GCUCAUCGCAAGAGUAGCATT	UGCUACUCUUGCGAUGAGCTT
c-Myc-homo-1344	GAGGAUAUCUGGAAGAAAUTT	AUUUCUUCCAGAUAUCCUCTT
c-Myc-homo-1729	GCUUGUACCUGCAGGAUCUTT	AGAUCCUGCAGGUACAAGCTT
c-Myc-homo-1982	GGAAGAAAUCGAUGUUGUUTT	AACAACAUCGAUUUCUUCCTT
Negative control	UUCUCCGAACGUGUCACGUTT	ACGUGACACGUUCGGAGAATT
