# Supplementary material for: Verdinexor, a Selective Inhibitor of Nuclear Exportin 1, Inhibits the Proliferation and Migration of Esophageal Cancer via XPO1/c-Myc/FOSL1 Axis
Source: Int J Biol Sci. 2022 Jan 1;18(1):276–91. doi: 10.7150/ijbs.66612 (PMC8692140; doi:10.7150/ijbs.66612)

Supplementary Figures

Figure Legend

Figure S1. Determination of drug concentration in wound width. Cells were seeded at a density of 12,000 cells/well in 96-well plates. Then cells were treated with KPT-335 from 0.3125 to 5 μM for 24 h and cell viability was performed by CCK-8 assay in KYSE30 and KYSE450. The data were analyzed by Student's t-test (two-sided) or one-way ANOVA. Error bars represent ± s.d., *$P < 0.05$, **$P < 0.01$.

Figure S1

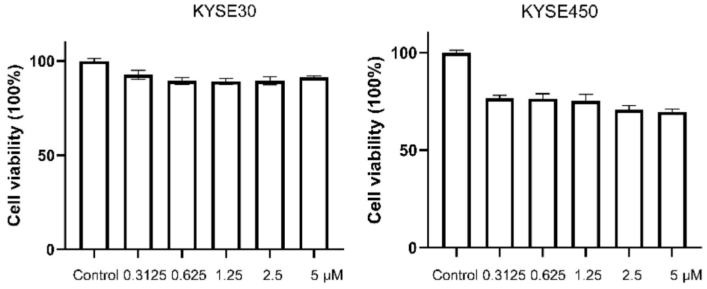

Supplement: Supplementary file 3 — Supplementary figure. [file ijbsv18p0276s3.pdf]
